# Gendered specialities during medical education: a literature review

**DOI:** 10.1007/s40037-014-0132-1

**Published:** 2014-07-01

**Authors:** Margret Alers, Lotte van Leerdam, Patrick Dielissen, Antoine Lagro-Janssen

**Affiliations:** Unit Gender and Women’s Health, Department of Primary and Community Care, Radboud University Medical Centre, ELG-117, PO Box 9101, 6500 HB Nijmegen, the Netherlands

**Keywords:** Medical students, Gender differences, Speciality preference

## Abstract

The careers of male and female physicians indicate gender differences, whereas in medical education a feminization is occurring. Our review aims to specify gender-related speciality preferences during medical education. A literature search on gender differences in medical students’ speciality preferences was conducted in PubMed, Eric, Embase and Social Abstracts, and reference lists from January 2000 to June 2013. Study quality was assessed by critical appraisal. Our search yielded 741 hits and included 14, mostly cross-sectional, studies originating from various countries. No cohort studies were found. Throughout medical education, surgery is predominantly preferred by men and gynaecology, paediatrics and general practice by women. Internal medicine was pursued by both genders. The extent of gender-specific speciality preferences seemed related to the male-to-female ratio in the study population. When a population contained more male students gynaecology seemed even more preferred by women, while in a more feminine population, men more highly preferred surgery. Internationally, throughout medical education, gender-related speciality preferences are apparent. The extent might be influenced by the male-to-female ratio of a study population. Further research of the role of gender in career considerations of medical students on the future workforce is necessary.

## Introduction

The increase in the proportion of women in medical schools suggests equal educational and professional opportunities [1, 2]. Interestingly, in the current medical profession the distribution of physicians across some specialities does not increase proportionally. There is an unbalanced horizontal segregation, exemplifying a vast majority of men surgeons and women gynaecologists [3–6]. Also disproportionately few women occupy senior positions in medicine, this is called vertical segregation [6, 7]. Gender seems to affect medical career choices.

Gender-related differences in medical career choices can be explained by several factors. Firstly, the cultural background might be an intrinsic influence on speciality choices. For example, women anticipate having a family and are thus probably more likely to choose a caring profession [8, 9]. Secondly, different choices in medical careers might be caused by gender bias. This might be the case in unequal treatment in educational opportunities and expectations or when negative experiences (gender discrimination or sexual harassment) in speciality orientation occur [5, 6]. On the other hand, some studies suggest that social behaviour of men and women is equal and not constraining. They see gender as one of multiple identities, that should be seen in context and the influence of gender should not be overrated [10]. Even though gender-related priorities of medical students do not appear of practical importance regarding motivation or skills, horizontal and vertical gender differences in medical careers have been indicated [6, 11]. Therefore, it is important to look at how women and men develop their career considerations during medical training.

At the start, both sexes receive equal access to medical education. During training, several factors lead to a particular medical speciality choice including gender [12]. In this study, we explore what is already known about gender-related speciality preferences during medical education. The aim is to [1] explore the extent of differences between speciality preferences of women and men medical students during the whole medical study including clerkships and [2] how women and men modify or remain with their speciality preferences.

## Method

### Search

A search strategy was formulated in PubMed and adapted for use in the databases of Eric, Embase and Sociological Abstracts (“Appendix 1”). A skilled librarian verified our search. Other relevant studies were collected by a hand search for references in all included articles (snowball method). No other additional searches were performed, e.g. via Internet search.

Because of diverse international denomination, medical students during the whole medical study were searched as: medical students, medical education and medical school. In the Netherlands a Bachelor and Master Degree structure is applicable [13]. At the European level, this structure has been introduced in medical curricula on a limited scale [14]. Terms for a bachelor degree were further defined as bachelor, undergraduate(s) and pre-graduate(s). Students before completion of their master degree programme were included using the keywords: master, internship, clerkship, house officer, foundation year, senior year and clinical rotation. Not included were graduates from medical school or medical physician, resident, registrar, senior house officer, fellowship, clinical attachment. For this review, we also used a gender filter, locating sex-specific evidence on clinical questions which has been adapted to PubMed [15]. The gender filter included keywords as gender, sex and differences. The primary outcome of studies included in our review was speciality preferences, also searched for as career choice.

### Inclusion and exclusion criteria

We searched the databases on articles published between 2000 and June 2013. The search included full-text studies of original research written in Dutch, English, French or German and published in peer-reviewed journals.

We included all studies meeting the following criteria: (1) involving medical students up till graduation, (2) assessing and reporting gender differences, and (3) evaluating speciality preferences for men and women. We excluded studies that (1) involved students or physicians in postgraduate training. As a result, general studies on career preferences were mostly not suitable. We also excluded studies (2) investigating the preference for a particular speciality or evaluating speciality preferences either for women or men solely.

### Selection and quality assessment

All review steps were performed by two reviewers independently (MTA, LL). We selected articles based on titles and abstracts. If agreement could not be reached between the reviewers on basis of title and abstract, the full-text article was assessed for eligibility.

Most selected articles concerned observational cross-sectional studies. There are few tools in the literature available to assess quality in observational studies [16] and only one of them had some interface with the selected articles in our review [17]. We assessed the quality of these quantitative observational cross-sectional studies using relevant critical appraisal criteria from other studies and based on Cochrane’s criteria [17–21]. Components included in our critical appraisal were [1] an evaluation of the appropriateness of the study design for the research question [2], a careful assessment of the key methodological features of the design [3], the appropriateness of statistical analysis, and [4] the legitimacy of conclusions (“Appendix 2”).

We included a component rating and a global rating for each article. Criteria were checked whether satisfied with a yes, can’t tell or no. When satisfied, 1 point was assigned. A total number of 10 points could be obtained for the individual criteria and these were proportionally distributed as [1–3] weak [4–6], moderate, and [7–10] strong. Both reviewers assessed reliability of the checklist in a pilot phase before applying it to all the selected studies. Ratings from the two researchers were averaged and studies with a quality score of seven points or higher were included in this review. Cohen’s Kappa was calculated of the reviewers’ applicability judgment to determine inter-rater reliability (good if >0.8, poor if <0.20) revealing a score of 0.87.

### Data extraction

We collected all possible specialities and compared main specialities across the studies between male and female students at the beginning and the end of their education. We described gender differences in speciality preferences for surgery, gynaecology including obstetrics, paediatrics, internal medicine and general practice. Anaesthesiology, dermatology, emergency medicine, ophthalmology, orthopaedics, psychiatry, radiology and other specialities were only described if of interest because these specialities are generally not preferred by large proportions of undergraduate medical students. When processing the results, we used the term male-to-female ratio to indicate the proportion of the number of male versus female students in the population. If there were more male students we named this ‘male-dominated’, while a study population with predominately female students was described as ‘female-dominated’.

## Results

**Fig. 1:**
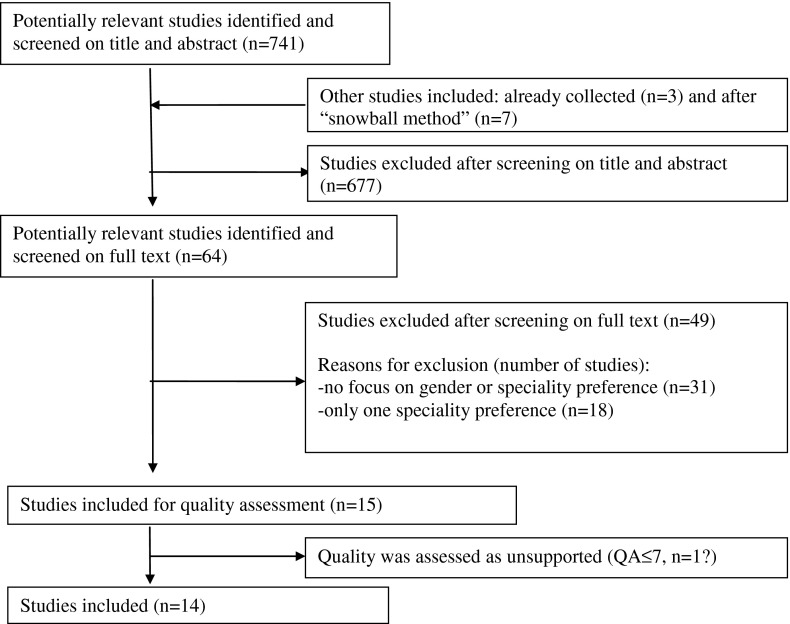
Flow chart of selection procedure

Figure 1 shows a flow diagram of the results of the selection process. We identified 741 articles of which 64 met our inclusion criteria. Most retrieved studies were excluded on the basis of title and abstract. After reviewing the full-text article we excluded 49 articles, leaving 15 articles for quality assessment. Quality assessment was not supportive for one study [22], thus 14 articles remained for data extraction [23–36] (Table 1).

**Table 1:** Characteristics and main findings of the included studies

Author (ref.)	Design	QA	Research question	Country	Year	M (n)	F (n)	Ratio M/F	Main findings
Al-Mendalawi [1]	Survey, cross-sectional	7	To study speciality preferences of medical students	Iraq	6	70	38	1.8	The most preferred clinical specialities chosen by male students were surgery (25 %), internal medicine (20.6 vs. 8.8 %) and paediatrics (16.2 vs. 8.8 %), whereas female students preferred gynaecology (19.1 vs. 1.5 %)
Compton [2]	Survey, cohort	9	Examine the association of speciality selections and change patterns with gender	USA	1, 2/3, 4	501	441	1.1	Of females starting with a preference for a non-primary care (non-PC) speciality (surgery, emergency room), 73 % remained in that category, compared with 90 % of males (*p* = .008). Females interested in PC (general practice, internal medicine, gynaecology, paediatrics) at three time points were 57, 41, and 44 %, compared with 34, 17, and 21 %, for males
Diderichsen [3]	Survey, cross-sectional	8	Investigate associations between motivational factors and speciality preference	Sweden	6	157	215	0.7	Both men and women preferred surgery, general practice and internal medicine most and gynaecology and paediatrics were also rather common. Almost a third of the students were uncertain of their speciality preference. Gynaecology was more often chosen by women graduates
Finucane [4]	Survey, cross-sectional	7	The career plans of interns	Ireland	6	134	165	0.8	Compared with men, women had twice as much interest in general practice, radiology, paediatrics, gynaecology and public health medicine and vice versa, men had a twofold preference for a career in surgery and anaesthetics
Fukuda [5]	Survey, cross-sectional	7	Investigate speciality preference in medical students	Japan	1-6	303	190	1.6	Internal medicine showed the highest preference rate, followed by general surgery, paediatrics, and emergency medicine. There was no significant correlation between the preference rates of men and women (r = 0.27, *p* = 0.34). The preference rates for general surgery, orthopaedics, neurosurgery, and emergency medicine were significantly higher in men than in women, while those of obstetrics and gynaecology, paediatrics, and dermatology were significantly higher in women
Fysh [6]	Survey, cross-sectional	8	Career intentions of first-year students and whether females prefer surgery or other specialities	UK	1	115	185	0.6	Males represented over two-thirds of the students wishing to pursue a career in surgery. Females intended to pursue a career in general practice and paediatrics. Two-fifth of both genders opted for internal medicine or had no preference yet
Hojat [7]	Survey, cross-sectional	8	Examine the relationships between speciality interest and gender	USA	1	517	559	0.9	Males were more interested in surgical specialities (64 vs. 36 %), whereas females comprised a larger proportion (65 vs. 35 %) of those interested in PC (general practice, internal medicine, paediatrics). The association between speciality interest and gender was statistically significant
Khader [8]	Survey, cross-sectional	8	To investigate the career preferences of medical students	Jordan	2,4,6	280	160	1.8	Most male students preferred surgery (52 %) compared with 15 % of female students (*p* < 0.005). Gynaecology was preferred by 31 % of female students compared with 1 % of male students. Males (15 %) and females (14 %) were equally likely to express interest in internal medicine
Ku [9]	Survey, “cohort”	7	Assess gender segregation across specialities for a cohort of physicians from their entry into medical school	USA	1,5	6,308	4,291	1.5	Speciality aspirations at entry into schooling are just as gender-different as speciality choices at exit. At entry gender gap in favour of females are gynaecology, paediatrics, general practice and in favour for males are surgical specialities
Lefevre [10]	Survey, cross-sectional	9	Speciality choice of medical students in sixth year of study	France	6	422	698	0.6	Gender influenced the choice of speciality: 88 % of future paediatricians, 82 % of gynaecologists and 77 % of general practitioners (GPs) were women (*p* < 0.05)
Mwachaka [11]	Survey, cross-sectional	10	Factors influencing choice of career in paediatrics	Kenya	1–5	217	168	1.3	Female students were five times more likely than males to select paediatrics
Mehmood [12]	Survey, cross-sectional	7	Determine variation in speciality preferences during medical school	Saudi Arabia	1–5	348	202	1.8	The most preferred speciality expressed by male students was surgery, followed by internal medicine and orthopaedics, while most preferred by female students were surgery, followed by paediatrics and ophthalmology
Parsa [13]	Survey, cross-sectional	7	Freshmen versus interns’ speciality interests	Iran	1–6	92	136	1.1	Female students showed little interest in surgery and most favoured specialities were gynaecology, paediatrics and internal medicine
Van Tongeren [14]	Survey, cross-sectional	8	Gendered speciality preferences of new medical students’	the Netherlands	1	188	428	0.7	40 % of both male and female students reported no speciality preference. Female students opted for paediatrics (19.2 %), whereas male students were more interested in surgery (25.5 %). None of the male students opted for gynaecology

### Specification studies

All included studies had a cross-sectional design and therefore could provide an answer to our first research question. Our search yielded no cohort studies which could draw conclusions on development in preferences. The participation rate of students in all included studies was 65 % or higher. The number of participants per study varied considerably from 38 to 4,291 female students and from 70 to 6,308 male students.

We included five studies from Europe [25, 26, 28, 32, 36], three studies from the United States [24, 29, 31], one study from Africa [34], four studies from the Middle-East [23, 30, 33, 35] and one study from Asia [27].

Seven studies evaluated students’ speciality preferences only once [23, 25, 26, 28, 29, 32, 36], five studies assessed speciality preferences twice [27, 31, 33–35] and two studies assessed students’ speciality preferences at three moments [24, 30].

Six studies reported gender differences at the start [24, 28–31, 36], two studies evaluated halfway medical education [24, 30], another six studies found evidence at the end [23, 25, 26, 30–32] and four studies gave an indication during the whole medical study [27, 33–35].

### Specialities more preferred by women

At the start of their medical education, women were especially interested in gynaecology and paediatrics. A preference for gynaecology was mentioned among 4–18 % of female students compared with 0–2 % of male students, for paediatrics this was 10–21 versus 2–9 % [24, 28–31, 36]. Women also opted for general practice more often than men (F 2–15 % vs. M 0–10 %) [24, 28–31, 36].

Halfway through their medical education, women showed a persistent interest in gynaecology (F 21 % vs. M 0 %), paediatrics (F 11 % vs. M 7 %) and general practice (F 4 % vs. M 1 %) [24, 30].

In addition, at the end of their medical education women continued to prefer a career in gynaecology (F 3–28 %, M 1–5 %), paediatrics (F 7–28 %, M 1–16 %) and general practice (F 0–21 % vs. M 2–17 %) [23, 25, 26, 30–32]. Two studies indicated the opposite; namely, that more male students chose paediatrics [23] or general practice [30] in comparison with female students. Studies following speciality preference throughout the medical education also found women mostly pursued a career in gynaecology (F 5–26 %, M 0–4 %) and paediatrics (F 6–24 %, M 3–7 %) [27, 35], though in one study more male students were interested in gynaecology (F 10 %, M 13 %) [34].

### Specialities more preferred by men

In five studies, surgery was the most frequently preferred speciality among men at the start of their medical education, but women showed an interest in surgery as well (F 10–25 % vs. M 39–64 %) [28–31, 36]. The interest of male students for surgery as speciality remained (F 9 % vs. M 55 %) [30]. One study from Sweden reported that an equal amount of women and men opted for surgery (F 17 % vs. M 23 %) [25].

By the end of medical education surgery was still the first choice of men (F 0–12 % vs. M 15–34 %) [23, 26, 30–32]. Several studies indicate that throughout medical education especially male students wished to pursue a career in surgery (F 10–17 % vs. M 26–35 %) [27, 33–35].

At the start, orthopaedics was also slightly more popular to men (F 6 % vs. M 8 %) [30, 33]. One study confirmed this midway (F 0 % vs. M 5 %), one at the end of medical education (F 0 % vs. M 13 %) [30]. Two studies confirmed men’s continuous interest in orthopaedics (F 2 % vs. M 7 %) [27, 33].

### Specialities preferred by both women and men

In three studies, at the start male and female medical students showed an equal interest in internal medicine (F 6–24 % vs. M 6–24 %) [28, 31, 36]. In one study male students were slightly more interested (F 3 % vs. M 8 %) [30]. Midway, one study confirmed an ongoing mutual interest in internal medicine (F 26 % vs. M 21 %) [30]. At the end of medical education internal medicine remained the largest equally chosen speciality (F 8–20 % vs. M 9–21 %) [25, 30, 31]. Yet, one study indicated it as a female speciality (F 14 % vs. M 8 %) [32], and one as a male speciality (F 9 % vs. M 21 %) [23]. In studies throughout the course, internal medicine remained a speciality preference for both male and female students (F 7 % vs. M 7–10 %) [27, 33–35].

### No speciality preference

There were no gender differences in students who had no speciality preference at the start (F 1–41 % vs. M 1–39 %) [28, 31, 36] or at the end of the medical curriculum (F 1–41 % vs. M 1–39 %) [25, 26, 31]. One study mentioned that men more often had no preference than women (F 15 % vs. M 23 %) [33].

### Influence male-to-female ratio on speciality preferences

In most studies with more male students than female students, i.e. ‘male-dominated’, women to a greater extent preferred gynaecology [23, 27, 30, 31, 33], whereas in a study population with predominately female students, ‘female-dominated’, still substantially more women chose gynaecology but to a lesser extent [26, 28, 32, 35, 36]. This tendency was also seen in paediatrics [26–28, 30–36] and general practice [23, 26–28, 31, 32, 34–36].

The opposite was seen in studies with a high male-to-female ratio, ‘male-dominated’, where substantially more men preferred surgery [23, 27, 30, 31, 33]. In ‘female-dominated’ study populations, proportionally a larger number of men opted for surgery [26, 28, 32, 35, 36].

There was no influence of the male-to-female ratio in internal medicine speciality preferences [28, 30–36].

## Discussion

We found that specific gender-related speciality preferences are present in the core choices of medical students from the beginning till completion of training, irrespective of nationality or country studied. In particular we saw this in surgery, a speciality highly attractive to men as well as in gynaecology, paediatrics and general practice, specialities which were mostly preferred by women. Internal medicine has an equal attraction to both women and men.

Medical students of both genders are potentially interested in various specialities. Female students are as likely as male students to start their career prospect in surgery but this preference decreases at the end of training, possibly due to heavy workload and a desire to have children [7, 28, 37]. The initial and final speciality preferences of men in our review seem more consistent than those of women [7]. Our results show gender differences in entering specialities at the start of medical careers.

It is challenging to compare study results of so many different countries and cultures. Discrepancies in the gender proportions selecting a speciality may also relate to the country of the study. The cultural background of each country should be taken into account to explain results. Differences in origins of studies might have societal implications. In the included papers, either women or men had the majority in a given speciality preference. In our mostly cross-sectional data it seemed that an unbalanced male-to-female ratio was associated with an even more disproportional selection of already gendered specialities. As such, the extent of gender differences in speciality preferences may relate to the male-to-female ratio in the study population. To determine the influence of the male-to-female ratio in a study population on speciality preferences, more research is needed. Possibly a meta-analysis on (preferably) cohort data in either male ‘dominated’ or female ‘dominated’ study populations could be helpful.

### Strengths and limitations

Strong points in our literature review are the reproducible and international search strategies with which we found sufficient studies of quality to answer our first research question. However, the number of articles found for inclusion may be a limitation, foremost in our finding on the influence of male-to-female ratio in a study population on speciality preferences. As the search only yielded English-language publications, publication bias could not be ruled out. Most studies were conducted at one university and therefore it might not have been representative of all medical students in that country. And although we critically appraised our studies, we might have paid too little attention to geographical distribution of the studies. Furthermore, our rating system for quality assessment could have produced other lists of articles than with other criteria or other weights.

### Interpretation and implications of findings

The increasing number of female students ensures a balance shift between the sexes in the medical profession and will weigh the importance of gender-differences in speciality preferences. Specialities such as gynaecology will be able to provide women patients with even more women gynaecologists. Therefore, it may be not necessary for faculty to reconsider access in single disciplines. However, male and female physicians are equally competent. Gender mainstreaming, which represents the process that brings gender issues from marginal into the core business of an organization, will offer institutions the opportunity to integrate a gender perspective into all phases of its programme cycle [38]. If the male-to-female ratio in specialities is unbalanced, possibly no new role models will be found [39].

Most of the studies call for better career advice by raising awareness about specialities earlier in education or for flexible work and training structures that allow work-life balance [7, 24, 27, 28, 30, 32, 34]. Medical education should include the choice of speciality from an early stage, so a future doctor, woman or man, can have an informed speciality choice on content. We should give more attention to how medical students come to their speciality preferences in order to anticipate how medical education can guide them.

We propose that policy makers take responsibility in matters of gender equality and gender equity when it comes to speciality distribution, instead of waiting till there is an intrinsic change in society in which this normal value is adopted. Reducing gender bias during studies adds value to medical training.

## Conclusion

We note that throughout undergraduate training in various countries some speciality preferences are specifically elected by women or men. Surgery is predominantly preferred by men and gynaecology, paediatrics and general practice by women. The extent of gendered speciality preferences seems related to the male-to-female-ratio in the study population.

Female or male students’ career choice seems to be a spontaneous or natural processes in medicine and our findings show that gendered speciality preferences are present throughout medical education. Given the current feminization it is important to pay attention to gender-related speciality preferences.

## Appendix 1: Search strategies

*PubMed (includes Medline)* ((((“students, medical”[MeSH Terms] OR medical students OR “education, medical”[MeSH Terms] OR medical education OR “schools. medical”[MeSH Terms] OR medical school)) AND (“Education. Medical. Undergraduate”[Mesh] OR “Young Adult”[Mesh] OR (“pre”[tiab] AND graduate*[tiab]) OR pregraduat* OR undergraduate* OR bachelor* OR master* OR internship and residency[MeSH Terms] OR internship OR clerkship OR house officer* OR foundation year* OR senior year* OR clinical rotation*)) AND (“gender identity”[MeSH Terms] OR gender OR “sex characteristics”[MeSH Terms] OR “sex differentiation”[MeSH Terms] OR sex differences OR sex differentiation OR “sex factors”[MeSH Terms] OR sex factors OR sex stereotypes OR (“equal” AND “opportunities”))) AND (“specialization”[MeSH Terms] OR specialization* OR “choice behavior”[MeSH Terms] OR choice* OR prefer* OR career*) AND (English[lang] OR French[lang] OR German[lang] OR Dutch[lang]) AND “2000/01/01”[PDAT] : “2013/06/10”[PDAT].

*Embase. Eric (OvidSP)* ((medical student or medical students or medical education or medical school).mp. or medical student/or exp Medical Education/or undergraduate*.mp.) and (gender.mp. or “equal opportunities (jobs)”/or sex role/or sex stereotypes/) and (specialization or career choice or prefer).mp limit to (dutch or english or french or german) and peer reviewed and yr = “2000–Current 2013”.

*Sociological abstract (CSA)* all(((medical AND (students OR student OR education OR school OR schools)) OR (undergraduate*) OR ((“medical students” OR “medical schools”))) AND ((gender) OR (gender difference*) OR ((“sex differences” OR “sex stereotypes” OR “sexual inequality”))) AND (((speciality OR career) AND (choice OR preference)) OR (specialization) OR ((“specialization” OR “occupational choice”)))) AND peer(yes) AND la.exact(“English” OR “Dutch” OR “French” OR “German”) AND pd(20000101-20130610).

## Appendix 2: Checklist of quantitative observational cross-sectional studies

When critically appraising a research article we tried to find answers to the following questions:

**Table:**

1	With regard to study design (three points): Was the study aim and research question or hypothesis clear? Was the motive or reason for the study stated? Was the study design appropriate for the research question?
2	With regard to the data collection (four points): Was the study sample clearly defined? In case of a sample, was the sample representative of the population? Is there an acceptable response rate (60 % or above)? Are the methods of data collection appropriate and explicitly described? E.g. consider whether (a) the variables were clearly defined and accurately measured and (b) measurements are justified and appropriate for answering the research question Did the study methods address the most important potential sources of bias? E.g. consider (a) selection bias (= an error in choosing the individuals or groups to take part in research) or (b) reporting bias (being more trusting of expected or desirable results, while under-reporting unexpected or undesirable experimental results)?
3	Statistical analysis (two points) Were the statistical analyses performed correctly? Is there a description of the statistical analysis with clarity of approach and credibility of the analysis: e.g. interpretations made by the researcher, how themes were derived?
4	Conclusions (one point) Do the data justify the conclusions?
